# Neurexin‐2 is a potential regulator of inflammatory pain in the spinal dorsal horn of rats

**DOI:** 10.1111/jcmm.15707

**Published:** 2020-11-08

**Authors:** Longsheng Xu, Qingli Feng, Housheng Deng, Xiaoping Zhang, Huadong Ni, Ming Yao

**Affiliations:** ^1^ Department of Anesthesiology and Pain Medicine The Affiliated Hospital of Jiaxing University (First hospital of Jiaxing) Jiaxing China

**Keywords:** inflammatory pain, neurexin‐2, proteomics, spinal dorsal horn

## Abstract

Chronic pain is one of the serious conditions that affect human health and remains cure still remains a serious challenge as the molecular mechanism remains largely unclear. Here, we used label‐free proteomics to identify potential target proteins that regulate peripheral inflammatory pain and reveal its mechanism of action. Inflammation in peripheral tissue was induced by injecting complete Freund's adjuvant (CFA) into rat hind paw. A proteomic method was adopted to compare the spinal dorsal horn (SDH) in peripheral inflammatory pain (PIP) model rats with controls. Differential proteins were identified in SDH proteome by label‐free quantification. The role of screened target proteins in the PIP was verified by small interfering RNA (siRNA). A total of 3072 and 3049 proteins were identified in CFA and normal saline (NS) groups, respectively, and 13 proteins were identified as differentially expressed in the CFA group. One of them, neurexin‐2, was validated for its role in the inflammatory pain. Neurexin‐2 was up‐regulated in the CFA group, which was confirmed by quantitative PCR. Besides, intrathecal siRNA‐mediated knock‐down of neurexin‐2 attenuated CFA‐induced mechanical and thermal hyperalgesia and reduced the expression of SDH membrane glutamate receptors (eg mGlu receptor 1, AMPA receptor) and postsynaptic density (eg PSD‐95, DLG2). These findings increased the understanding of the role of neurexin‐2 in the inflammatory pain, implicating that neurexin‐2 acts as a potential regulatory protein of inflammatory pain through affecting synaptic plasticity in the SDH of rats.

## 1 BACKGROUND

Chronic pain is one of the serious conditions that affect human health, and its cure remains a serious challenge as the molecular mechanism remains largely unclear. Inflammatory pain, one of the chronic pains, is generally caused by trauma, bacterial or viral infection, and peripheral tissue damage due to surgery,^1^ and this pain model can be used for understanding the mechanisms of the chronic pain. In this study, a single‐exposure inflammatory model around the joint was used, that is the complete Freund's adjuvant (CFA) was administered by injection into the plantar surface of the left hind paw to cause inflammation in the local tissue around the joint.^2^ After 1 day of CFA injection, the local area showed obvious redness, self‐protection, and movement disorder as well as mechanical and thermal allodynia.^3^ Also, symptoms of inflammatory pain were developed as hyperalgesia and allodynia.^4^ The alteration in the proteome that assists in identifying proteins was studied. It is critical to understand the mechanisms of inflammatory pain and the selection of novel drug targets.

With the rise and development of high‐throughput genomic and proteomic technologies, many new molecules have been developed and many disease‐related key targets and pathways have been revealed, providing an opportunity for developing new drugs.^5^ Proteomics becomes a research hotspot for studying various disease mechanisms.^6^ Gene function is realized by proteins. Proteins are regarded as real‐life activity performers and can better reflect the current functional state of the body. If the molecular mechanism that is associated with pain can be directly studied from the protein level, the essential act of chronic inflammatory pain will be revealed more comprehensively and systematically. Studying proteomics involves a study based on the proteome, analyses the changes in protein composition, level and modification status from the overall level, understands the interaction between proteins and reveals the function of proteins and the laws of cell activities.^7^

In this study, a proteomic‐based strategy was used to compare the spinal dorsal horn (SDH) in a peripheral inflammatory pain (PIP) model of a rat with a control group. PIP was induced by the injection of CFA unilaterally into the rat hind paw. Differential proteins were founded in the SDH proteome using label‐free quantification. Further investigation was conducted by bioinformatics analysis, and the changes of target proteins were verified by Western blotting and quantitative PCR (qPCR) methods. Furthermore, small interfering RNA (siRNA) was used to study the effect of target protein on inflammatory pain and its mechanism. Here, we demonstrate that neurexin‐2 is a regulator of inflammatory pain.

## 2 METHODS

### 2.1 Reagents and animals

The antibody information was listed in Table S1. Neurexin‐2‐siRNA for in vivo transfection and its mismatch control (MC‐siRNA) were purchased from RiboBio Co.^8^ Adult male Sprague Dawley rats (200 ± 20 g, n = 136) were housed in an SPF room with temperature of 21‐23°C and accessed food and water ad libitum. Animal experiments were approved by the Jiaxing University IACUC and were performed abiding with the ethical standards of the Helsinki Declaration and the International Association for the Study of Pain's guidelines for pain research in animals. The detailed methods were listed in Appendix S1.

### 2.2 Pain model establishment

The pain model was established according to the previously published method.^4^ Briefly, CFA was injected subcutaneously into rat left hind paw.

### 2.3 Drug administration

Intrathecal tubing and drug administration were conducted as reported previously.^9^ Neurexin‐2‐siRNA and MC‐siRNA were dissolved in saline at a final concentration of 2 nmol/μL (10 μL, n = 8) for subarachnoid administration before and on days 1 and 3 after the CFA injection.

### 2.4 Thermal paw‐withdrawal latency test

Thermal paw‐withdrawal latency (TWL) test was performed as described previously.^4^ The paw‐withdrawal latencies were recorded.

### 2.5 Mechanical paw‐withdrawal threshold test

Mechanical paw‐withdrawal threshold (MWT) test was performed as described previously.^4^ MWT was recorded.

### 2.6 Protein preparation

After treatment, L4‐L6 enlargement of left SDH was collected. The proteins were obtained from tissue homogenates in RIPA buffer (MO, Sigma). Homogenates were centrifuged at 13,000 *g* at 4°C for 10 min, and then, the supernatants were collected. Protein concentration was measured using the BCA protein assay kit (Pierce, Thermo Scientific). Two hundred fifty microgram protein of each sample was taken and digested according to previously described method^10^ for proteomic determination.

### 2.7 Proteomic determination and sequence database searching and data analysis

The protein samples were analysed by HPLC‐MS/MS according to the method in Appendix S1.

### 2.8 Screening siRNA sequence targeting rat neurexin‐2

The scrambled and neurexin‐2 siRNA1, siRNA2 and siRNA3 sequences and knock‐down experiments were described in Appendix S1.

### 2.9 Western blot analysis

Western blot analysis was described in Appendix S1. Briefly, proteins were separated by SDS‐PAGE, incubation of primary and second antibodies were conducted, and protein band was visualized using the ECL method.

### 2.10 Real‐time quantitative PCR

The qPCR was performed as in Appendix S1. ^ΔΔ^ *C*_t_ method was used to evaluate the differential expression.

### 2.11 Statistical analysis

All data are presented as mean ± SE. The differences in the molecular expression or behavioural scores among groups were analysed by one‐ or two‐way repeated‐measures analysis of variance, respectively, followed by the Bonferroni test. *P* values < .05 were considered to be statistically significant.

## 3 RESULTS

### 3.1 CFA induces hyperalgesia

**FIGURE 1:**
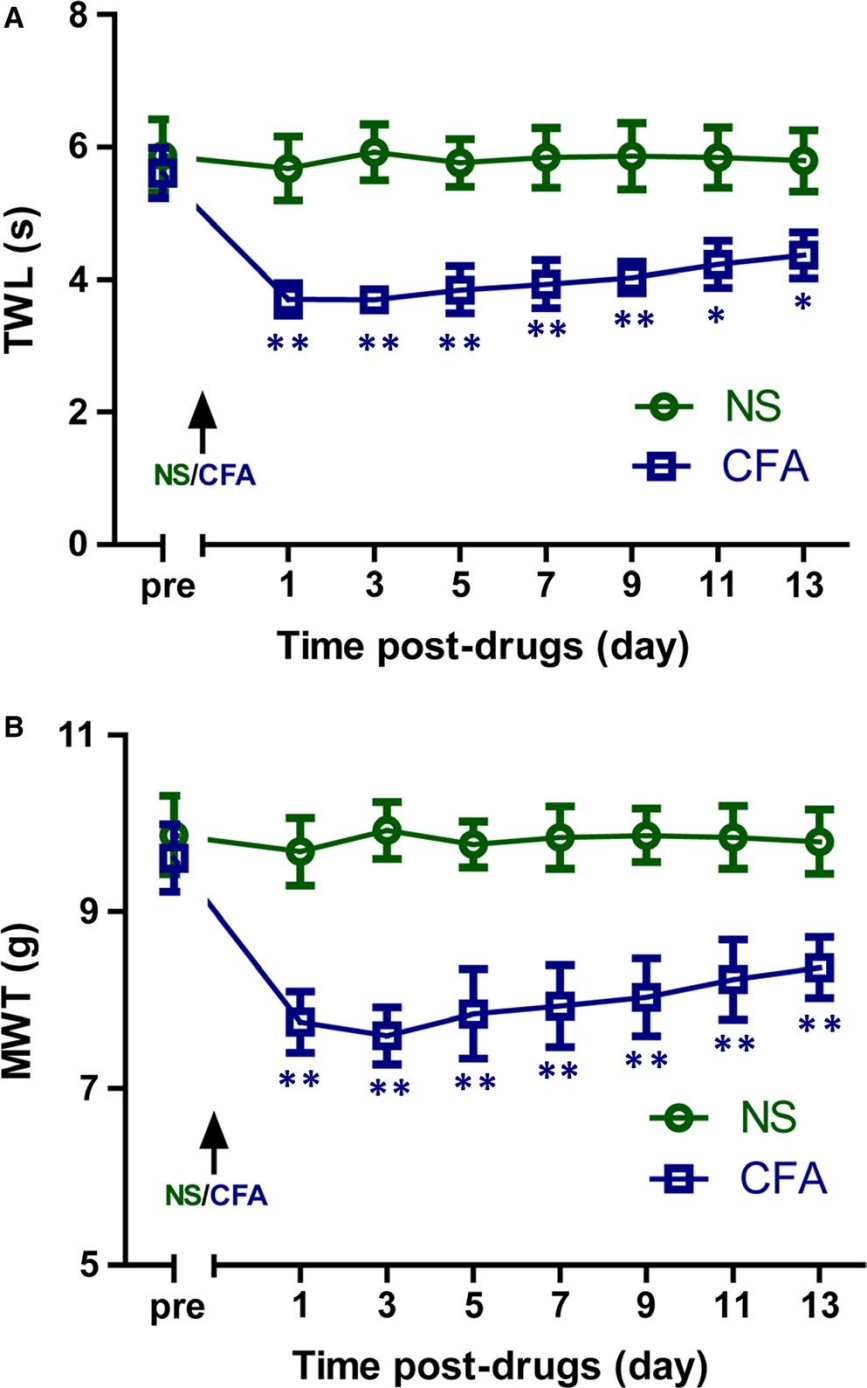
Complete Freund's adjuvant (CFA) produces inflammatory hyperalgesia in rats. Thermal withdrawal latency (TWL; A) and mechanical withdrawal threshold (MWT; B) were significantly decreased in CFA‐treated rats when compared with normal saline (NS)‐treated rats. Data are expressed as means ± SE (n = 8). *P < .05, **P < .01 vs NS‐treated rats

In the current study, a rat CFA model that was used in several studies was adopted for investigation. Figure 1 showed the behavioural effects of CFA and NS when injected subcutaneously into the left hind paw. MWT testing and TWL testing were performed on rats before and every 2 days (last for 13 days) after CFA administration. Before injection, the rats in the NS and CFA groups had similar values to the pain stimuli. The CFA rats had a significant reduction in the pain responses from the first day. This suggests behavioural signs of the inflammatory pain. The NS rats showed slight changes with no significant increase in mechanical and thermal responses (Figure 1A,B).

### 3.2 Overall protein changes identified by proteomics

**FIGURE 2:**
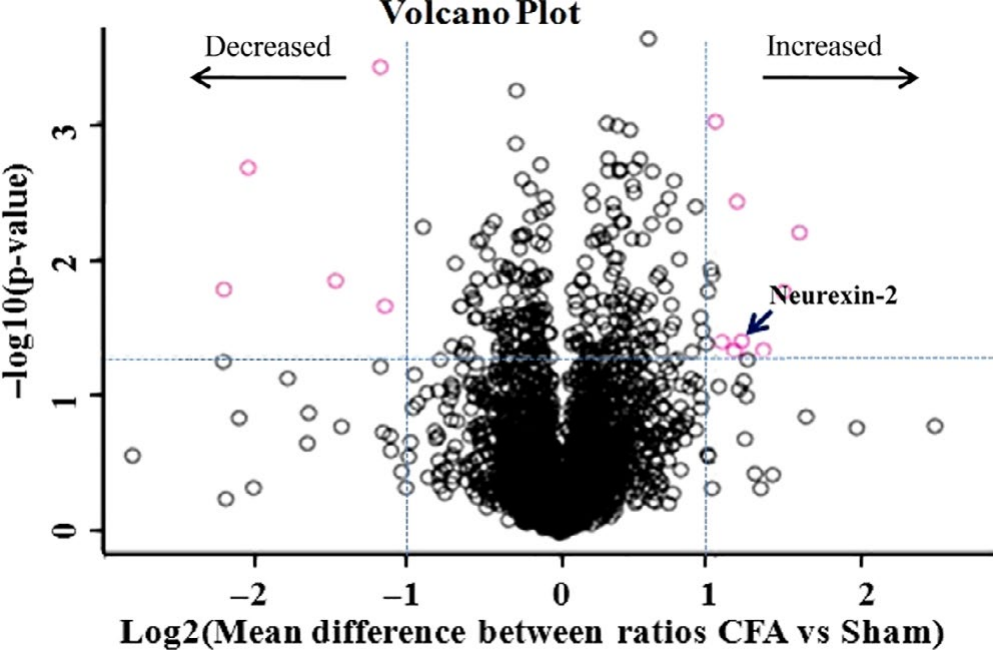
Identification of proteins that show significant changes in abundance after complete Freund's adjuvant (CFA) injection. Proteins with more than twofold differential expression data were analysed within PERSEUS using three‐sample*t*tests and a false discovery threshold of 0.05 to identify candidates that significantly change in abundance. Five proteins were down‐regulated, and eight proteins were up‐regulated in abundance 3 d post‐CFA injection relative to NS injection. “↙” marked as neurexin‐2

Using proteomics, a total of 3072 and 3049 proteins were identified in CFA and normal saline (NS) groups, respectively. Of them, 2992 proteins were identified in both groups. Thirteen proteins were found to be dysregulated between two groups (CFA/NS ratio ≥twofold or ≤0.5‐fold, *P*‐value < .05). Of these proteins, five were down‐regulated, and eight proteins were up‐regulated (Figure 2, Table 1).

**TABLE 1:** Differentially expressed proteins with more than twofold differential expression and *P*‐value < .05 in CFA rats vs NS rats identified from proteomics analysis

Protein IDs	Protein name	Gene name	AVG_CFA_	AVG_Sham_	CFA/Sham	t test *P*‐value
A0A0H2UHS7	60S ribosomal protein L18	Rpl18	760 986 666.7	379 913 333.3	2.0030533	.0009454
Q5PQU1	Kininogen 1	Kng1	155 840 000	70 695 000	2.2043992	.0036839
P08932	T‐kininogen 2	Kng2	663 346 666.7	228 196 666.7	2.9069078	.0062072
P06907	Myelin protein P0	Mpz	6 503 300 000	2 395 866 667	2.7143831	.0168115
D4A0W1	ER membrane protein complex subunit 4	Emc4	22 143 666.67	9 857 866.667	2.246294	.0389439
*D3ZAD6*	*Neurexin‐2*	*Nrxn2*	*19 773 000*	*9 587 100*	*2.0624589*	*.0395407*
A0A0G2K3C8	Nidogen‐2	Nid2	50 950 000	20 579 500	2.4757647	.0451098
P62634	Cellular nucleic acid‐binding protein	Cnbp	245 236 666.7	112 522 000	2.1794553	.045429
B6ID11	Ubiquitin‐related modifier 1	Urm1	42 588 666.67	94 911 666.67	0.448719	.0003747
Q6MG71	Choline transporter‐like protein 4	Slc44a4	7 390 800	30 368 500	0.2433706	.0020601
Q5M7V3	LOC367586 protein	LOC367586	373 328 000	1 039 316 667	0.3592052	.0139214
Q4V8A7	Atp8b2 protein	Ubap2l	19 949 300	91 604 333.33	0.2177768	.0161572
Q5UAJ6	Cytochrome c oxidase subunit 2	COX2	1 070 570 000	2 339 666 667	0.4575737	.0213484

The significance of italic formating implies that it is the target protein for further study in this article.

### 3.3 Bioinformatics analysis

**FIGURE 3:**
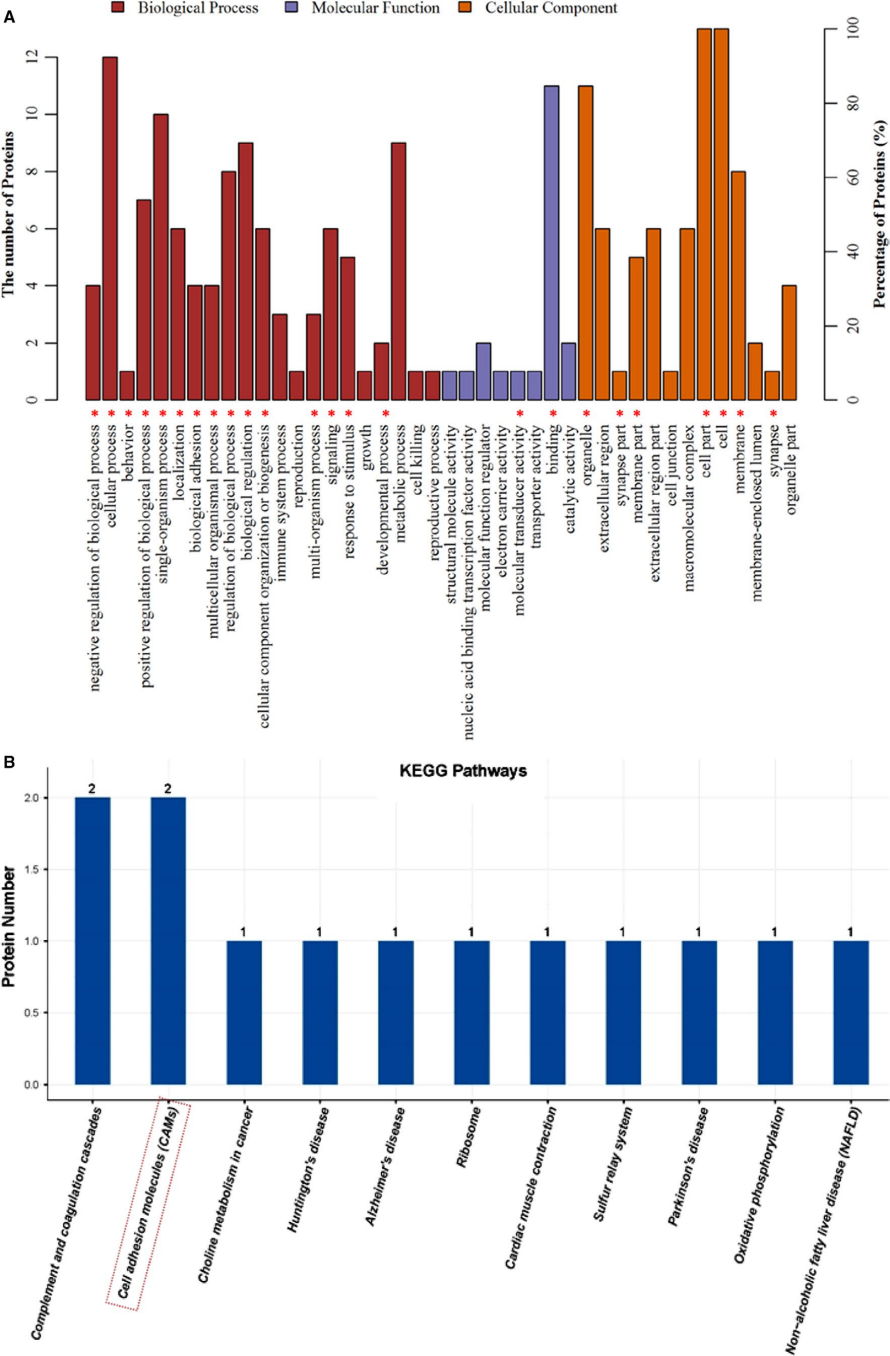
The functional annotation of dysregulated proteins was analysed by protein analysis. A, Biological process, molecular function and cellular component. B, Pathway analysis of 13 dysregulated proteins was indicated by PANTHER, DAVID, STRING and Reactome. “*,” involved neurexin‐2

Biological function analysis (GO analysis and pathway analysis) was performed on 13 different proteins obtained in this experiment by the DAVID enrichment analysis system. GO analysis of 13 differential proteins was performed to study their function, and biological process (BP) analysis results revealed that differentially expressed proteins are mostly involved in the cellular process, single‐organism cellular process, biological regulation and metabolic processes. Molecular function (MF) analysis revealed that most of these proteins are related to molecular binding. Cellular component (CC) analysis revealed that the differential proteins were mainly distributed in the cell membrane and organelles, and extracellular regions (Figure 3A). Among the 13 differential proteins, KEGG pathway showed two proteins each related to complement and coagulation cascades and cell adhesion molecules (CAMs), and one protein each related to choline metabolism in cancer, Huntington's disease, Alzheimer's disease, ribosome, cardiac muscle contraction, sulphur relay system, Parkinson's disease and oxidative phosphorylation (Figure 3B).

### 3.4 Identification of neurexin‐2

**FIGURE 4:**
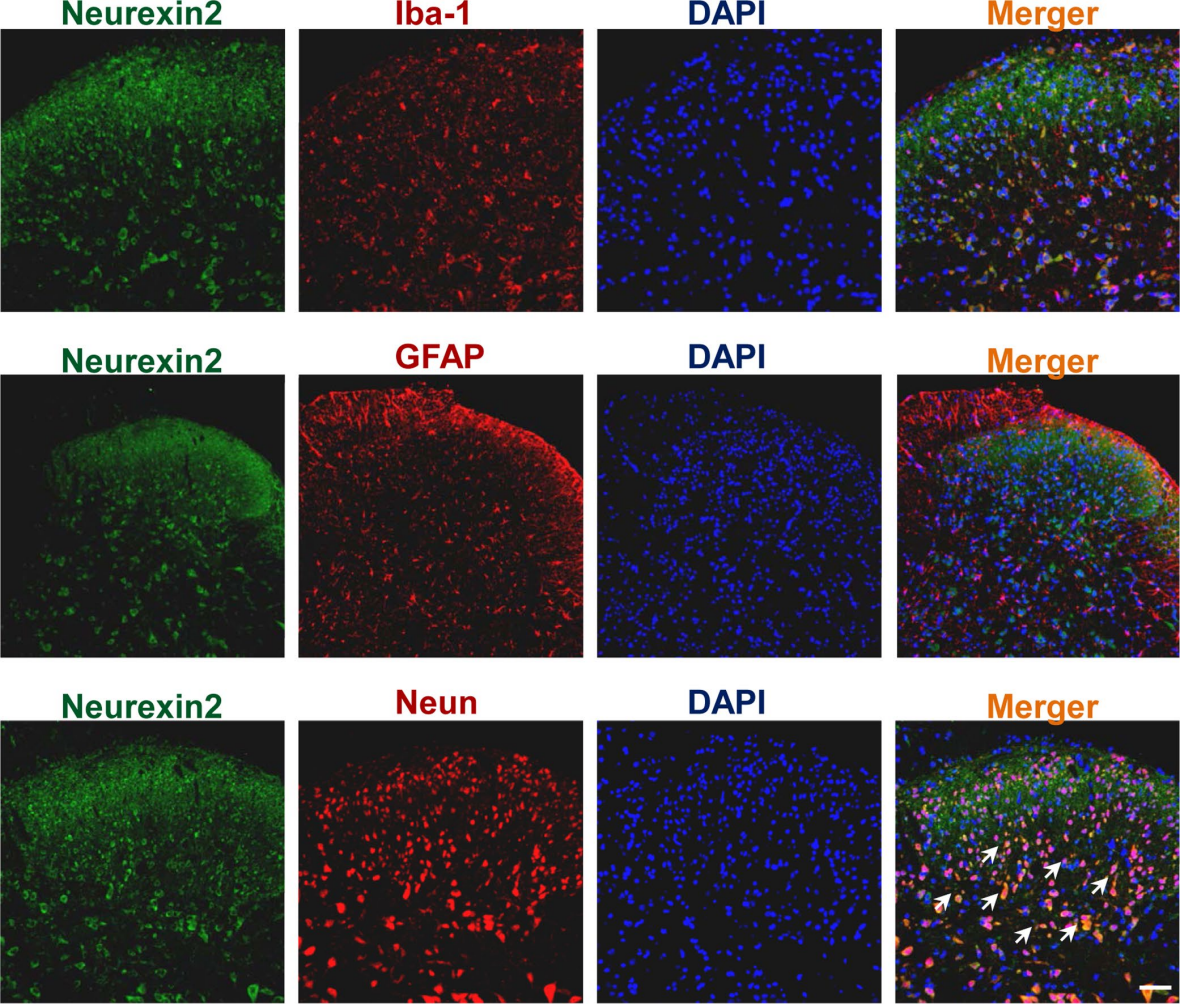
Immunofluorescence double staining showed that neurexin‐2 (green) was colocalized with neuronal marker, NeuN (red), but neither with astrocyte marker GFAP (red) nor with microglial marker Iba‐1 (red). Original magnification: 100×, scale bar: 100 μm

Through KEGG pathways, neurexin‐2 was found to be one of the CAMs among the 13 differentially expressed proteins (Figure 3B). Furthermore, in order to understand the cellular distribution of neurexin‐2, immunofluorescence double staining of neurexin‐2 with NeuN (neuronal marker), GFAP (astrocyte marker) or Iba‐1 (microglial marker), respectively, was performed. Immunofluorescence images showed that neurexin‐2 was colocalized with neuron, but neither with astrocyte nor with microglia (Figure 4). This suggests that neurexin‐2 is located in the neuron cell. In the BP, neurexin‐2 participates in biological regulation, biological adhesion and development process. Based on the MF, neurexin‐2 is mainly involved in molecular transducer activity and binding. In the CC, neurexin‐2 was distributed in the cell membrane and synaptic sites (Figure 3A). Furthermore, previous studies have reported that neurexin is mainly distributed in the pre‐synapses of neurons and is very important for the formation and differentiation of synapse by binding neuroligin that is expressed in the postsynaptic neurons.^11^ One of the main functions of neurexin is to regulate the growth of synapses.^12^ The changes of synaptic plasticity in the formation and maintenance of hyperalgesia have long been agreed^13^; however, the role of neurexin in hyperalgesia has not been reported so far, and hence, this study selected neurexin‐2 for further research.

### 3.5 Validation of neurexin‐2 by qPCR and Western blot

**FIGURE 5:**
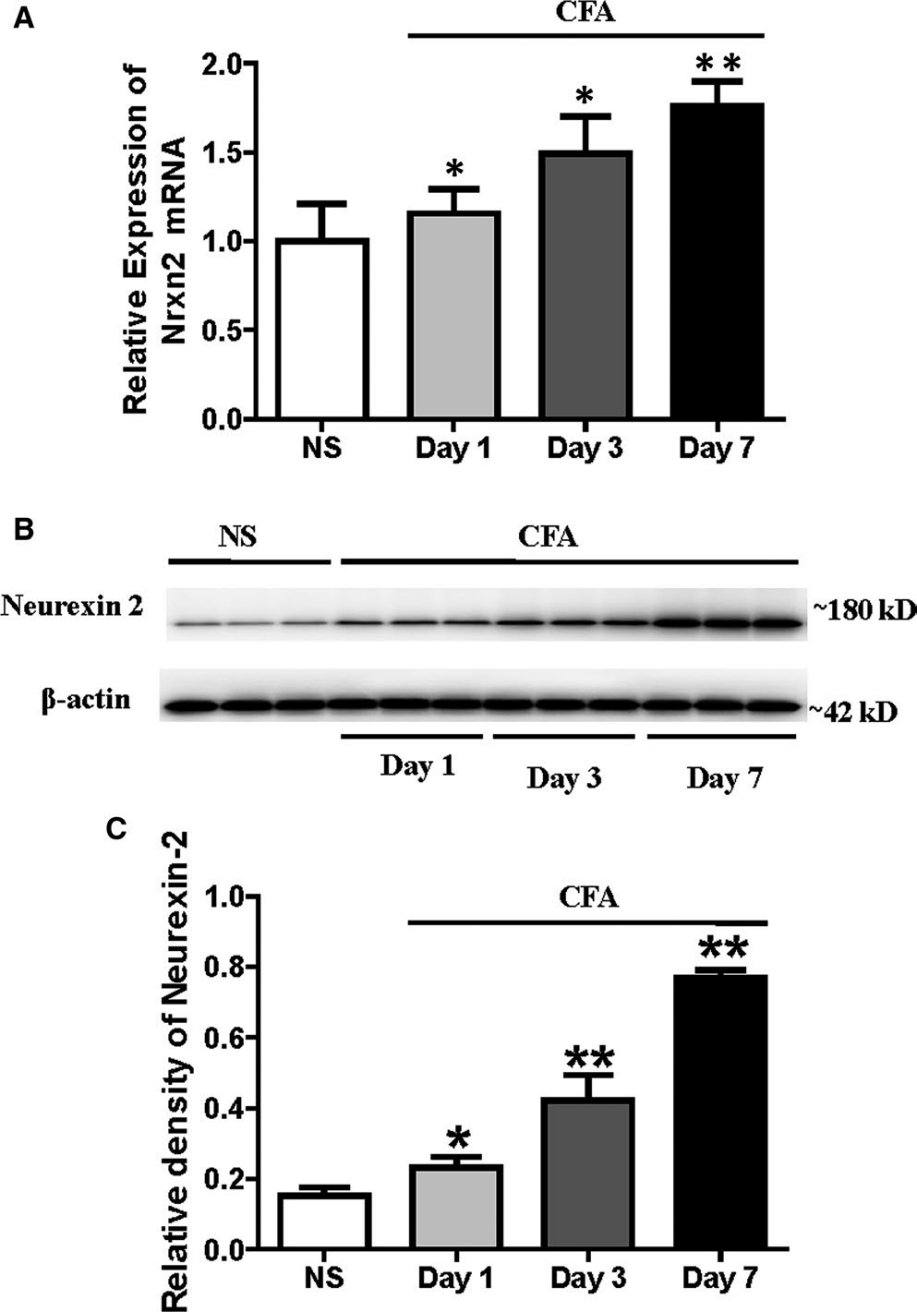
CFA induces a time‐dependent increase in spinal neurexin‐2 mRNA and protein expression. The expression of neurexin‐2 was assayed on days 1, 3 and 7 after CFA injection. A, qPCR results showed that the mRNA expression of *Nrxn2* was a time‐dependent up‐expression in SDH tissues from CFA‐treated rats (n = 6). B, Representative Western blots for neurexin‐2 and β‐actin at different time‐points after CFA injection; β‐Actin was used as the loading control. C, The bar graph showed that the relative level of neurexin‐2 protein was increased in SDH tissues of CFA‐treated rats in a time‐dependent manner (n = 6). *P < .05, **P < .001 vs NS rats

The differential expression of neurexin‐2 in the SDH tissue from NS and inflammatory rats was validated by qPCR and Western blotting. Both have demonstrated a significant up‐regulation of neurexin‐2 in the SDH tissues from CFA‐treated rats when compared to NS rats (Figure 5A‐C). These results all showed that neurexin‐2 was up‐regulated in the SDH tissue from inflammatory rats by qPCR, Western blotting and proteomics approach.

### 3.6 Silencing of the neurexin‐2 transgene with siRNA in neurons separated from SDH tissue

**FIGURE 6:**
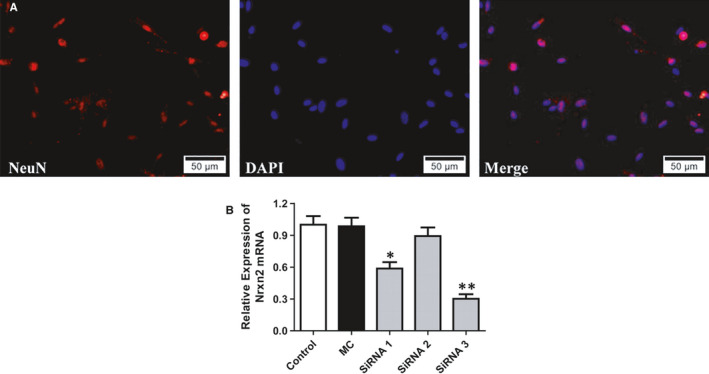
Screening siRNA for efficient suppression of *Nrxn2* expression in vitro. Neurons in SDH tissues were cotransfected with either one of the three independent siRNA oligonucleotides targeting *Nrxn2* (neurexin‐2‐siRNA1‐3) or a mismatch control siRNA (MC‐siRNA). Two days after transfection, the expression of *Nrxn2* mRNA was measured by qPCR. A, Identification of primary cultured neurons from the rat SDH tissues (×50 μm). B, Quantification of *Nrxn2* siRNA knock‐down was evaluated by qPCR. The results revealed that two siRNAs demonstrated efficient inhibition on *Nrxn2* mRNA, and neurexin‐2‐siRNA3 being the most potent one (n = 3). *P < .05, **P < .001 vs MC‐siRNA group

Three siRNA oligonucleotides (siRNA1, siRNA2 and siRNA3 sequences) targeting the rat neurexin‐2 were used to cotransfect in neurons from SDH tissue in vitro (Figure 6A) and then the level of neurexin‐2 transgene expression was analysed by qPCR (Figure 6B). The results of qPCR revealed that neurexin‐2‐siRNA1 and neurexin‐2‐siRNA3 have demonstrated effective inhibition, and neurexin‐2‐siRNA3 was the most potent one. Therefore, Neurexin‐2‐siRNA3 was used for further in vivo studies.

### 3.7 Intrathecal injection of selected neurexin‐2‐siRNA specifically decreases neurexin‐2 expression levels in the SDH tissue

**FIGURE 7:**
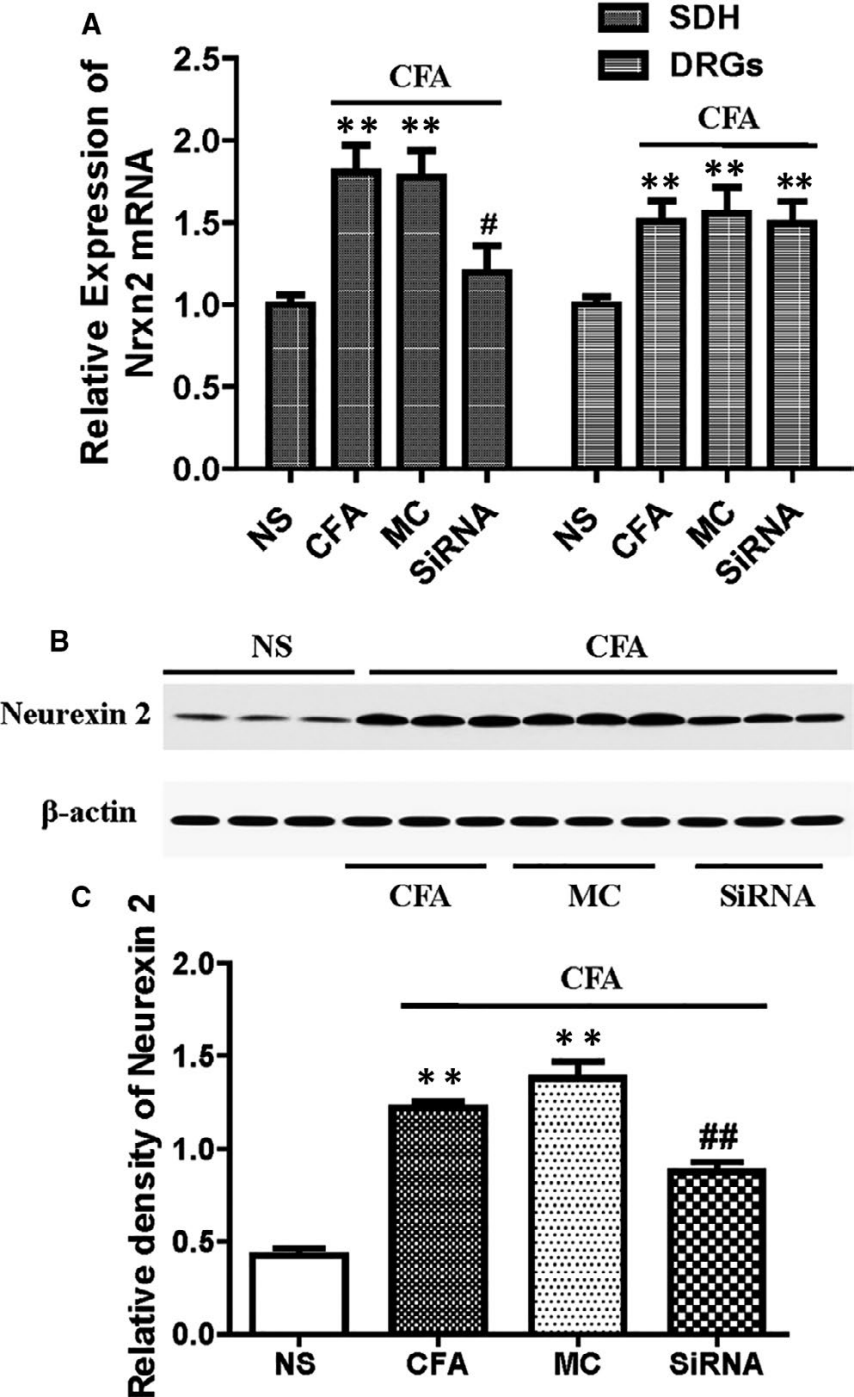
Complete Freund's adjuvant (CFA) increases *Nrxn2* mRNA and neurexin‐2 proteins in the dorsal horn of spinal cord L4‐6 segments and selected neurexin‐2‐siRNA that reverses this effect by intrathecal injection once in 2 d for 0‐4 d as described in the section2. A, qPCR analyses of *Nrxn2* mRNA expression in rat lumbar SDH tissues and dorsal root ganglions in four groups. B, Representative Western blots of neurexin‐2 and β‐actin at different administration reagents; β‐Actin was used as the loading control. C, Bar graph showed that the relative level of neurexin‐2 protein was increased in SDH tissues of CFA‐treated rats, and increased neurexin‐2 expression in the selected neurexin‐2‐siRNA treatment group was significantly lower than the MC‐siRNA compared with MC‐siRNA rats (n = 6). ^#^ *P* < .05 vs NS group; **P < .001 vs MC‐siRNA group

The effects of neurexin‐2‐siRNA on expression levels of neurexin‐2 in SDH tissues were also analysed via qPCR and Western blotting. As outlined in Figure 7A, following intrathecal injection of neurexin‐2‐siRNA, the expression of *Nrxn2* mRNA in SDH neurons was decreased when compared to the MC‐siRNA group (Figure 7A). Furthermore, Western blot was subsequently performed to compare the protein expression of neurexin‐2 in SDH tissues among the groups. Consistent with the mRNA results, intrathecal injection of neurexin‐2‐siRNA resulted in a 43.7% reduction of neurexin‐2 protein levels when compared with the MC‐siRNA control group (*P* < .05, Figure 7B,C).

### 3.8 Intrathecal injection of neurexin‐2‐siRNA reduces MWT and TWL in peripheral inflammatory pain rats

**FIGURE 8:**
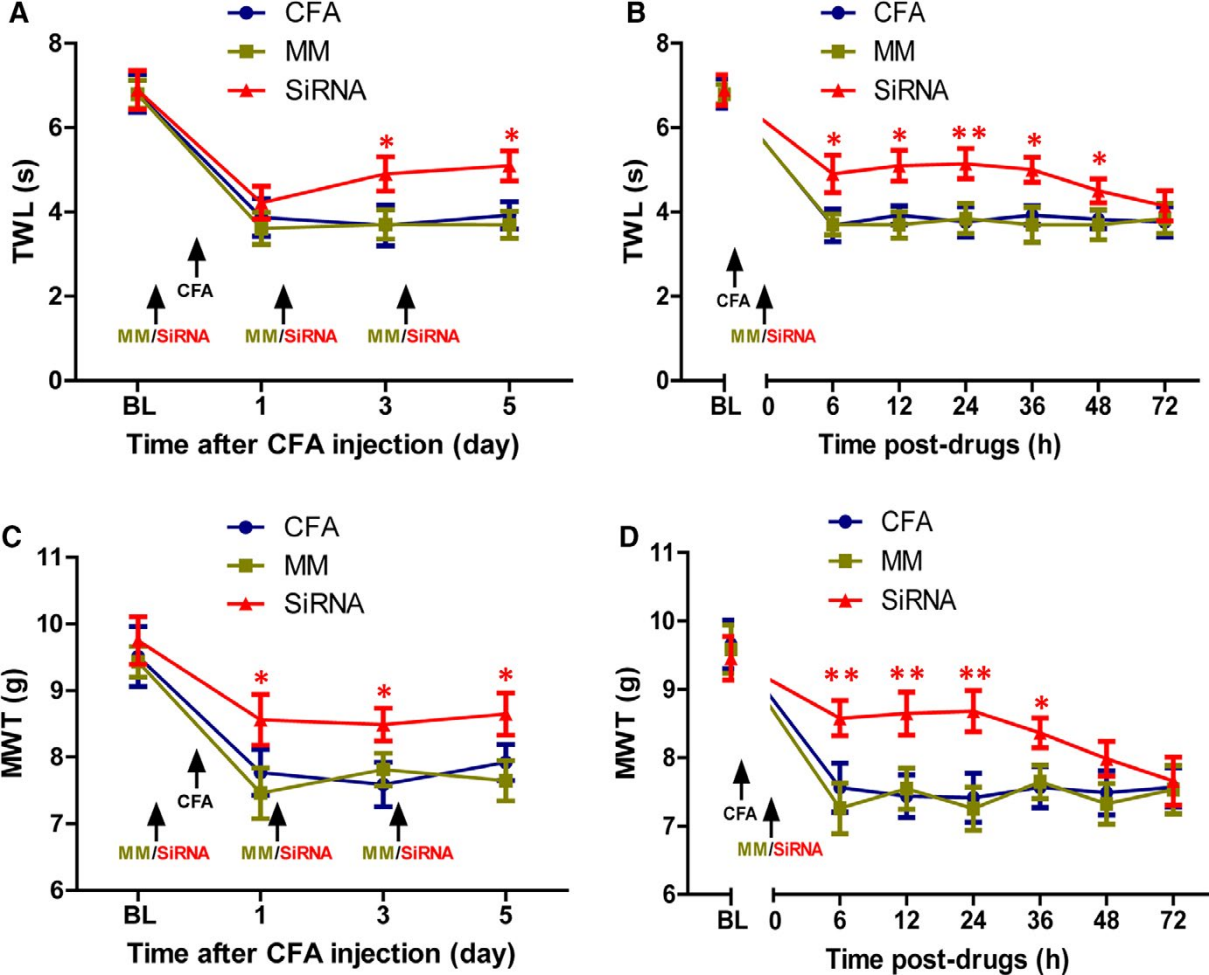
Inhibitory effects of neurexin‐2‐siRNA on tactile allodynia and thermal hyperalgesia induced by CFA. Neurexin‐2‐siRNA (20 nmol in 10 μL; n = 8) or MC‐siRNA (20 nmol in 10 μL; n = 8) was microinjected into subarachnoid haemorrhage before and on days 1 and 3 after CFA injection. A, C, MWT and TWL were used to measure mechanical allodynia and thermal hyperalgesia on days 1, 3 and 5 after drug administration, respectively. The MWT and TWL showed no change in CFA and MC‐siRNA groups (*P* > .05). In contrast, mechanical allodynia and thermal hyperalgesia induced by CFA was attenuated by intrathecal administration with neurexin‐2‐siRNA, but not mismatched siRNA (n = 8). B, D, After three times of the administration, the MWT and TWL were increased at 6 h, reached a peak at 24 h and lasted until 48 h compared with MC‐siRNA rats (n = 8). *P < .05, **P < .001 vs MC‐siRNA group

To evaluate the effects of neurexin‐2‐siRNA on CFA‐induced pain behaviours, MC‐siRNA or neurexin‐2‐siRNA was intrathecally administered before and on days 1 and 3 in the CFA group. The mechanical allodynia and thermal hyperalgesia were measured on days 1, 3 and 5 after CFA injection by MWT test and TWL test, respectively. The MWT and TWL results showed no changes in the CFA and MC‐siRNA groups (*P* > .05, n = 8/group). In contrast, the MWT and TWL of CFA rats were attenuated by intrathecal administration of neurexin‐2‐siRNA, but not mismatched siRNA (*P* < .05, n = 8/group, Figure 8A,C). Furthermore, after the last neurexin‐2‐siRNA treatment, compared to the MC‐siRNA group, the MWT and TWL results were increased at 6 hours, reached peak at 24 hours and lasted till 48 hours (*P* < .05, n = 8/group, Figure 8B,D). The results showed that neurexin‐2‐siRNA reduced thermal hyperalgesia and mechanical allodynia in peripheral inflammatory pain rats.

### 3.9 Intrathecal injection of neurexin‐2‐siRNA reduces the expression of membrane glutamate receptors and postsynaptic density protein in the SDH tissue

**FIGURE 9:**
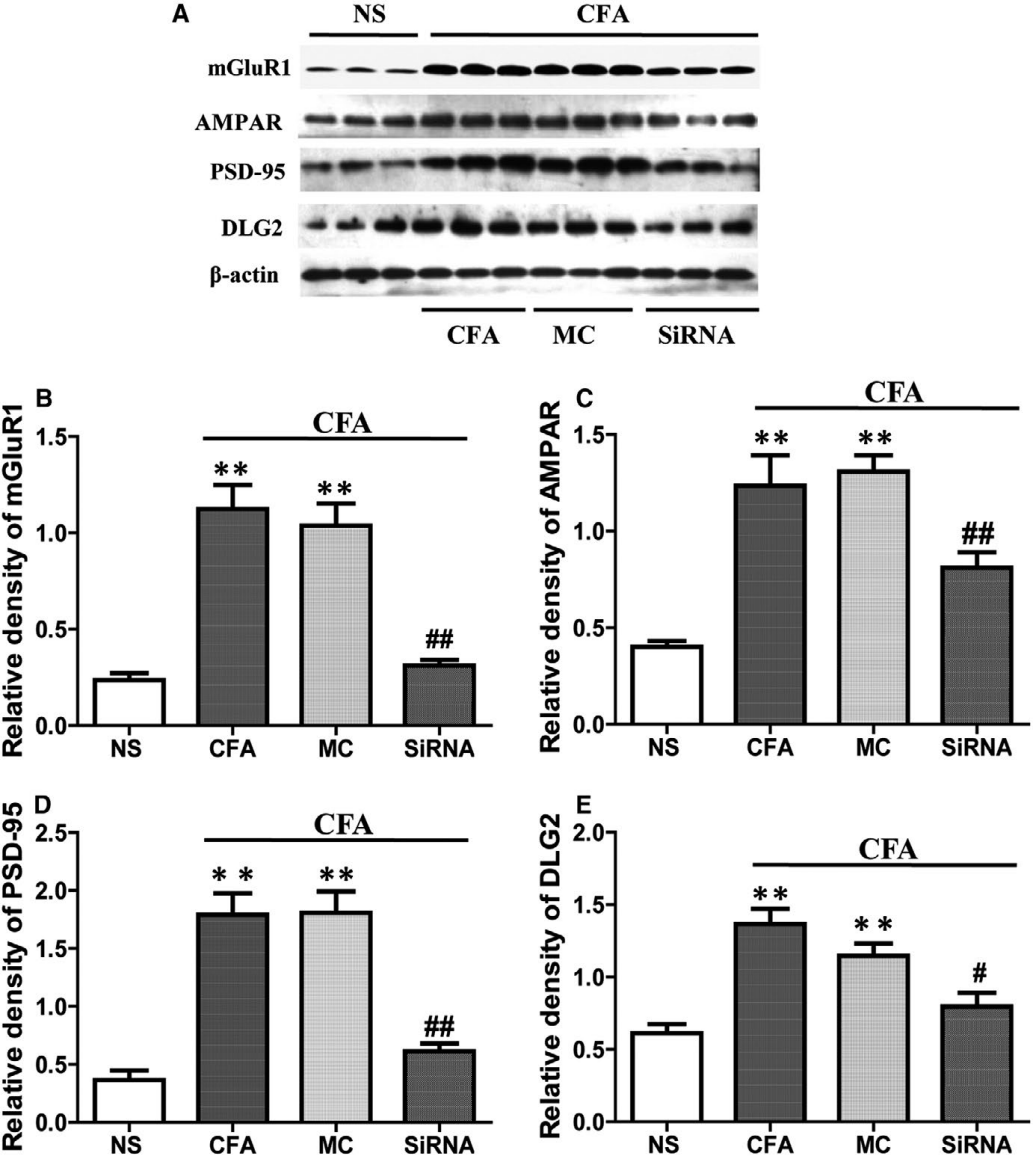
Complete Freund's adjuvant (CFA) increases the expression of mGlu1 receptor, AMPA receptor, PSD‐95, DLG2 membrane proteins in the dorsal horn of spinal cord L4‐6 segments, while neurexin‐2‐siRNA reverses this effect by intrathecal injection as described in the section 2. A, Representative Western blots of mGlu1 receptor, AMPA receptor, PSD‐95, DLG2 membrane proteins and β‐actin with different administration reagents; β‐Actin was used as the loading control. B‐E, Bar graph showed that the relative level of mGlu1 receptor, AMPA receptor, PSD‐95 and DLG2 membrane proteins was increased in SDH tissues of CFA‐treated rats, and increased proteins in the neurexin‐2‐siRNA treatment group were significantly lower than the MC‐siRNA (n = 6). **P < .001 vs NS group; ^#^ *P* < .05, ^##^ *P* < .001 vs MC‐siRNA group

Combining with the previously studied effects of neurexin‐2 with the mechanism of peripheral pain, this study probed the effect of neurexin‐2 on the expression of membrane glutamate receptors and postsynaptic density (PSD). Western blot results showed that glutamate receptors mGlu receptor 1, AMPA receptor and PSD proteins PSD‐95 and DLG2 on the membrane were increased in SDH tissue of CFA rats, but no differences were observed in four proteins between CFA‐treated rats and MC‐siRNA rats. However, compared with the MC‐siRNA group, the expression of four proteins on membranes from in the SDH tissue was significantly lower in the CFA group after 5 days of CFA treatment (*P* > .05, n = 6/group, Figure 9A‐E). This indicated that intrathecal administration of neurexin‐2‐siRNA reduced the expression of membrane glutamate receptors and PSD in the SDH tissues.

## 4 DISCUSSION

Complete Freund's adjuvant is a potent inflammatory agent that causes localized and long‐lasting inflammatory pain. In this study, the rat model of chronic inflammatory pain was prepared by injection of CFA 0.1ml in the hind paw as described in the literature.^14^ The results showed that MWT was decreased on days 1, 3 and 7 after CFA injection. The shortening of TWL suggests that the rat model of chronic inflammatory pain was successfully prepared and is regarded as an ideal research object in comparative proteomics research of chronic inflammatory pain. SDH is the gateway to sensory information and an integrated primary centre.^15^ Peripheral noxious stimulation can be directly transmitted to SDH neurons for integration through dorsal root ganglion axons.^16^, ^17^ Therefore, in our study, the global proteomic changes in SDH of a CFA rat model using quantitative proteomics method coupled to bioinformatics analysis was focused. A total of 3129 proteins were quantified in both CFA and NS rats. There were 34 differentially identified proteins based on a *P*‐value of <.05 or 39 differentially identified proteins based on the CFA/NS ratio of ≥twofold or ≤0.5‐fold. However, there were only 13 differentially identified proteins based on the CFA/NS ratio of ≥twofold or ≤0.5‐fold and *P*‐value of <.05. The reason for such a low number of proteins differentially identified is unclear. This may be because the tissue sample detected in this experiment is the dorsal horn of the spinal cord, which is far away from the site of inflammation and there is a barrier between blood and brain. Peripheral inflammation leads to changes in proteins that are difficult to enter the back of the spinal cord.

Bioinformatics analysis combined with the related literature was used to predict the pain‐related protein and selected neurexin‐2 for further research. Neurexin‐2 expression was validated by Western blotting and qPCR in the SDH of CFA model rats. Consequently, *nrxn2* and neurexin‐2 were increased along with the up‐regulation of PSD and glutamate receptors. Furthermore, intrathecal injection of neurexin‐2‐siRNA in CFA rats inhibited the pain threshold in them, and the up‐regulation of PSD and glutamate receptor expression in the SDH endometrium was also partially inhibited. These findings suggested that neurexin‐2 may be pertinent to CFA‐induced inflammatory pain and may contribute to the understanding of pathogenic mechanisms, thereby providing evidence on novel therapeutic targets for inflammatory pain.

Our findings revealed that neurexin‐2 expression showed a significant increase in SDH of CFA rats when compared with the control group. While massive efforts have been made in recent years to investigate the molecular mechanisms of synaptic levels within the CNS, very little has been reported on neurexin‐2 in SDH of CFA rat models.^18^, ^19^ Neurexin‐2 is one of the neurexins that cleave isomers, and are presynaptic proteins that help to connect neurons at the synapse.^20^, ^21^ They contain a single transmembrane domain and primarily located on the presynaptic membrane. The extracellular domain interacts with proteins in the synaptic cleft, most notably neuroligin, while the intracellular cytoplasmic portion interacts with proteins associated with exocytosis.^21^

Neurexin/neuroligin (the receptor of neurexin) is encoded by *nrxn*/*nlgn* genes, respectively. Mutations in *nrxns*/*nlgns* are associated with a variety of neuropsychiatric disorders, suggesting the importance of its function.^22^ Since its first discovery in 1992, research on *nrxns*/*nlgns* has been carried out for more than 20 years, and the function of *nrxns*/*nlgns* in mammals has been extensively explored. In 2000, Scheiffele et al^23^ found that *nlgns* are artificially expressed on axons forming a presynaptic structure in non‐neuronal cells. In 2004, Graf et al found that transfection of *nlgn* into COS cells induced excitatory synaptic vesicle molecules VGlut1, and the inhibitory synaptic vesicle molecule GAD65 is recruited in adjacent axons, whereas the expression of *nrxn* alone in COS cells sufficiently induced recruitment of excitatory postsynaptic protein PSD‐95 and the inhibitory postsynaptic protein gephyrin. This confirmed that the LNS domain mediates this process, indicating that neurexin can induce post‐synaptic differentiation.^24^ Further experiments demonstrated that neurexin recruits post‐synaptic proteins by binding to neuroligin. In 2005, Chih et al have revealed that the expression of *nlgns* in mouse hippocampal neurons culture promoted post‐synaptic differentiation in vitro. When RNA interference was used to down‐regulate the expression of *nlgns*, the excitatory synaptic and inhibitory synapses were significantly reduced, further confirming that *nlgns* can modulate the formation of synapses.^25^ In summary, neurexins mediate signalling across the synapse and influence the properties of neural networks by synapse specificity. Neurexin and neuroligin “shake hands,” resulting in a connection between the two neurons, promoting pre‐ and post‐synapse specialization in structural differentiation and being involved in the regulation of synaptic structures and functions. Neurexin‐2 might act as a potential regulatory protein by affecting the formation of synapses and reducing the expression of membrane glutamate receptors on hyperalgesia in CFA rats.

In conclusion, our present study suggests that injecting CFA into rat hind paw induced peripheral inflammation and pain hypersensitivity. In association with these changes, a total of 3072 and 3049 proteins were identified in CFA and NS rats, respectively, and 13 proteins were identified as differentially expressed in the CFA group by proteomics‐based strategy. Protein profile of the SDH in CFA rats was performed, which facilitates a comprehensive and comprehensive understanding of the mechanism of inflammation pain development. Combined with bioinformatics analysis and related literature, neurexin‐2, a differentially expressed protein was selected for validation, which was consistent with proteomic analysis. Intrathecal siRNA‐mediated suppression of neurexin‐2 attenuated CFA‐induced hyperalgesia and inhibited the expression of SDH membrane glutamate receptors (eg mGlu receptor 1, AMPA receptor) and PSD (eg PSD‐95, DLG2). These findings increased the understanding of neurexin‐2 in inflammatory pain, implicating that neurexin‐2 might act as a potential regulatory protein of inflammatory pain formation through affecting synaptic plasticity in the SDH of rats.

## CONFLICT OF INTERESTS

The authors confirm that they have no competing interests.

## AUTHOR CONTRIBUTION

**Longsheng Xu:** Data curation (lead); Methodology (lead); Project administration (lead). **Qinli Feng:** Methodology (equal); Project administration (equal); Writing‐original draft (equal). **Housheng Deng:** Methodology (equal). **Xiaoping Zhang:** Methodology (equal); Project administration (equal). **Huadong Ni:** Funding acquisition (supporting); Methodology (equal). **Ming Yao:** Data curation (equal); Funding acquisition (lead); Validation (lead); Writing‐original draft (lead); Writing‐review & editing (lead).

## ETHICS APPROVAL

All procedures were approved by the Committee on the Use of Live Animals in Teaching and Research and performed according to the guidelines for the care and use of laboratory animals as established by the Laboratory Animal Unit at the Jiaxing University.

## Supporting information

TableS1

AppS1

## Data Availability

The data that support the findings of this study are available in the supplementary material of this article.
