# Supplementary material for: Neurexin‐2 is a potential regulator of inflammatory pain in the spinal dorsal horn of rats
Source: J Cell Mol Med. 2020 Nov 8;24(23):13623–33. doi: 10.1111/jcmm.15707 (PMC7754071; doi:10.1111/jcmm.15707)
Supplement: Supplementary file 1 — TableS1 [file JCMM-24-13623-s001.docx]

**Supplementary Table S1. Antibody information**

| **Antibody name** | **Cat. no** | **Company (City, State)** | **Dilution** |
| --- | --- | --- | --- |
| PSD-95 | 3450 | Cell Signaling Technology (Danvers, MA) | 1:1000 |
| DLG2 | 19046 | Cell Signaling Technology (Danvers, MA) | 1:1000 |
| mGlu receptor 1/5 | SAB5200492 | Sigma Chemical Co (St. Louis, MO) | 1:1000 |
| AMPA receptor 1 | SAB4501293 | Sigma Chemical Co (St. Louis, MO) | 1:1000 |
| Neurexin-2 alpha | ab34245 | Abcam Company (Cambridge, UK) | 1:800 |
| β-Actin | [SAB5500001](https://www.sigmaaldrich.com/catalog/product/sigma/sab5500001?lang=zh&region=CN) | Sigma Chemical Co (St. Louis, MO) | 1:10,000 |
