# Supplementary material for: Neurexin‐2 is a potential regulator of inflammatory pain in the spinal dorsal horn of rats
Source: J Cell Mol Med. 2020 Nov 8;24(23):13623–33. doi: 10.1111/jcmm.15707 (PMC7754071; doi:10.1111/jcmm.15707)
Supplement: Supplementary file 2 — AppS1 [file JCMM-24-13623-s002.docx]

**Supplementary material S1**

# 2.2 Pain model establishment

For the induction of inflammatory pain, CFA (50% in saline, with 5 mg/ml heat-killed mycobacterium tuberculosis, 0.1 ml) was injected subcutaneously into rat left hindpaw. Symptoms of inflammatory pain such as redness, swelling of the ankle joint, hyperalgesia, and impairment in motor activity were evident after 1 day of CFA injection. Normal rats were injected witha similar volume of normal saline (NS), as controls[[1](#_ENREF_1)].

# 2.3 Drug administration

As reported previously[[2](#_ENREF_2)], anintrathecal catheter (PE-10 microtube) was inserted into the intervertebral space between L4 and L5 for the drug delivery. The back of the rats was cleanly shaved and the rats are placed briefly under anesthesia using pentobarbital sodium (60 mg/kg, i.p.). When PE-10 microtube was inserted into the intervertebral disc and extended into the subarachnoid space of the lumbar enlargement, the microtube was then fixed to the adjacent ligament by 3-0 suture with 2cm microtube free-end exposed and plugged. Next, the surgical incision was sutured. The day after surgery, paralysis of the two hind limbs after intrathecal delivery of 10 μl lidocaine along the tube indicated successful catheterization. On day 2 after the operation, the two legs that are paralyzed by intrathecal injection of lidocaine again indicated a successful insertion. Drugs were administered by a connection of 20-μL micro-syringe pre-filled with a drug to the catheter. Neurexin-2-siRNA and MC-siRNA were dissolved in the physiological saline at a final concentration of 2 nmol/μL (10 μL, n=8) for subarachnoid administration before and on days 1 and 3 after CFA injection.

# 2.4 Thermal paw-withdrawal latency (TWL) test

All pain behavioral studies were carried out in a quiet room from 9:00 AM to 11:00 AM. Rats were allowed to habituate to the environment for 30 min before the experiments have begun. TWL test was used to determine hyperalgesia during subsequent measurements.The tail-flick unit (Ugo Basile) was used[[3](#_ENREF_3)], in which the rats were gently held, and the left hindpaw pad waskept over the flush-mounted window containing the heat source set at 50°C. The paw withdrawal latencies were defined as the time taken by the rat to remove its hindpaw from the heat window. The cut-off point was set at 10 s to prevent tissue damage. All pain thresholds were measured thrice with an interval of 1–2 mins and the average was taken as TWL(s).

# 2.5 Mechanical paw-withdrawal threshold (MWT) test

MWT was tested as follows. Rats were gently held and an incremental pressure (maximum 25 g) was applied onto the dorsal surface of the left hindpaw using the Analgesy-Meter (Ugo Basile) as described previously [[4](#_ENREF_4)]. The pressure required to elicit paw withdrawal was determined as MWT (in grams). The cutoff point was set at 25 g to prevent tissue damage. In such a case, 25 g was determined as the MWT. The pressure-bearing spot of the dorsal surface was marked to make sure that the repeated-measured MWT was from the same point, and the average values were taken as MWT (g).

# 2.6 Protein preparation

After treatment, L4–L6 left enlargement of the SDH from CFA and control groups rats (three rats per group) was rapidly collected and stored in liquid nitrogen. The whole proteins were obtained from tissue homogenates in RIPA buffer (MO, Sigma), followed by centrifugation for 10 min at 13,000 g at 4°C, and then the supernatants were collected. After protein concentration was determined by BCA (bicinchoninic acid) protein assay kit (Pierce, Thermo Scientific, Germany), protein aliquots were stored at -80°C.Protein (250 μg for each sample) digestion was performed according to the FASP procedure described by Wisniewski, J. R., et al[[5](#_ENREF_5)]. Briefly, the detergent, DTT, and other low-molecular-weight components were removed using 200 μl UA buffer (8 M Urea, 150 mM Tris-HCl pH 8.0) by repeated ultrafiltration (Microcon units, 30 kD) facilitated by centrifugation. Then, 100 μL 0.05 M iodoacetamide in UA buffer was added to block the reduced cysteine residues and the samples were incubated for 20 min in darkness. The filter was washed with 100 μl UA buffer thrice and then 100 μl 25 mM NH4HCO_3_ twice. Finally, the protein suspension was digested with 3μg trypsin (Promega) in 40 μl 25 mM NH4HCO_3_ overnight at 37°C, and the resulting peptides were collected as a filtrate. The peptide content was estimated by UV light spectral density at 280 nm using an extinction coefficient of 1.1 of 0.1% (g/l) solution, which was calculated based on the frequency of tryptophan and tyrosine in vertebrate proteins.

# 2.7 Proteomic determination-- Label free quantification

To quantify the proteins from the LC-MS/MS, a label-free quantification analysis was performed using PEAKS Studio 8.0 (Bioinformatics Solutions Inc., Waterloo, ON, USA) ([Zhang et al., 2012](https://hub.pubmedplus.com/10.1074%2Fmcp.m111.010587)). Independent samples from each triplicate analysis were studied and compared between CFA and control groups rats. Total raw data files were imported and processed using the Peaks software program for the interpretation of spectra and the retention time was set from 600 to 10,500 s. An in-house constructed Uniprot’s reference database of *Homo sapiens* (release 03_2014) contained 20,272 entries was added and combined with a decoy database (the sequences were reversed). For label-free quantification the following parameters were specified: enzymatic digestion by trypsin, with two missed cleavages; precursor mass tolerance was 10 ppm; fragment mass tolerance: 0.7 Da, minimum charge: 2, maximum charge: 3. The specified fixed and variable modification consisted of carbamidomethylation (Cys), oxidation (M), and deamidated (N and Q). To determine the false-positive identification rate, the estimated spectra was used against decoy database. A false discovery rate (FDR) of ≤1%, with a peptide score of −10 log *p* ≥ 20 was considered adequate for confident protein identification. To determine the relative protein and peptide abundance in the tested samples, peptide feature based quantification was performed. The signal intensity of a peptide is directly proportional to the abundance of the peptide in the sample, therefore, the confidently identified peptide features were matched and the peptide intensity differences between two samples were able to be estimated. Likewise, the area under the curve of the extracted ion chromatograms (XICs) was measured and compared between two analyzed runs. To get the summed cumulative peak area of the protein, only unique peptides that are assigned to particular proteins were selected.

The FDR was calculated based on the target/decoy database, and the peptides with an FDR of ≤1% were chosen as true positive hits (considering the risk of having one false positive in 20 observations). By using this active feature based quantitative approach the detected peptides with *p*-values <0.05 and 0.01 which were identified in at least three observations from the SLE samples compared to control samples were considered. In order to identify the significant protein differential expressions an independent sample *T*-test was performed. The quantified datasets were normalized using their spectral abundance factor values (the average of the triplicate experiments) and this was used to generate a heat map showing the differentially expressed proteins between the two groups.

# 2.8 Screening siRNA sequence targeting rat neurexin-2

A scrambled sequence was designed as a mismatch control (5’-UCGCCUGAACUCUAGCUGA-3’). SiRNAs targeting cDNA sequence of rat neurexin-2 (GenBank accession NM_ 053846) were as follows: 5’- GGUCCUUACUGUUCUCCGAdTdT-3’ (siRNA1), 5’-CGCUCAGCACCGUCAAGUAdTdT-3’ (siRNA2), 5’-CGACGAGGGCUCCUACCAAdTdT-3’ (siRNA3). All siRNAs were chemically synthesized by Ribobio Co. (Guangzhou, China) [[7](#_ENREF_7)]. To identify the knockdown efficiency of different siRNA oligonucleotides, primary cultured mouse SDH neurons were co-transfected with neurexin-2-siRNA (SiRNA1-3, respectively) using lipofectamine 2000 (Invitrogen, USA). An oligonucleotide sequence with no homology to the sequence of *Nrxn2* was used as mismatch controls. After 48 h, the inhibitory effects of different siRNA oligonucleotides were determined by measuring *Nrxn2*expression using Real-time quantitative PCR (qPCR). The siRNA fragment with the highest inhibition rate was selected and administered (i.t.) into the L4-L6 SDH of the rat after 5’Chol + 2’ OMe modification.

# 2.9 Western blot analysis

The prepared proteins were separated by SDS-PAGE using Criterion XT Precast 6% Bis-Tris gels (Bio-Rad, Hercules, CA) and then were electro-transferred onto PVDF membranes (Invitrogen) in standard transfer buffer (25 mM Tris, 192 mM glycine, and 10% vol/vol methanol, pH 8.3) for 1.5 h at room temperature. After the membranes were blocked with 5% nonfat milk in TBS and 0.1% Tween-20 for 1 h, the bound proteins were exposed to specific antibodies against PSD-95 (1:1,000), DLG2 (1:1,000), mGlu1 receptor 1/5 (1:1,000), AMPA receptor 1 (1:1,000), neurexin-2 alpha (1:800), and β-actin (1:10,000, rabbit monoclonal; Sigma) for overnight at 4°C. After extensive washing in TBS and 0.1% Tween-20, a 1:5,000 dilution of goat anti-rabbit horseradish peroxidase secondary antibody (Jackson Immunoresearch) was used as appropriate and incubated for 1 h at room temperature. After extensive washing, the signals were detected using Western Lightning ECL and quantified relative toβ-actin control by densitometry on Image-Pro Plus 6.0.

# 2.10 Real-time quantitative PCR

QPCR was performed. PCR reactions were performed in a final volume of 20 μL containing 2× PCR master mix (Fermentas, K0171), sense and antisense primers, and diluted cDNA. The following primers were used to amplify *Nrxn2*: 5'- CGCTCTGCATCCTTATCCT TCTCTA-3’ (forward) and 5'- TCTTTGTCTTTGTTCTTCTTGGCCT-3’ (reverse). The annealing temperature was set at 53°C. qPCR amplification was performed using a Rotor-gene QTAMRA 1109 Sequence Detection System (QIAGEN Techservice) and the reaction conditions were as follows: 2 min at 95°C for polymerase activation and 95°C for 15 s, 53°C for 20 s, and 60°C for 30 s for amplification and signal collection. β-Actin was used to normalize the expression levels of target genes, and ^- Δ Δ^Ct method was used to evaluate the differential expression.

# 2.11 Statistical analysis

All data are presented as mean ± SE and are calculated using SPSS version 20.0. The differences in the molecular expression or behavioral scores among groups were analyzed by one-or two-way repeated measures analysis of variance,respectively, followed by Bonferroni test. *P* values < 0.05 were considered to be statistically significant.

1. **Xu L, Pan Y, Zhu Q, Gong S, Tao J, Xu GY, Jiang X.** Arcuate Src activation-induced phosphorylation of NR2B NMDA subunit contributes to inflammatory pain in rats. *Journal of neurophysiology*. 2012; 108: 3024-33.

2. **Wang BC, Hillman DE, Li D, Turndorf H.** Lumbar subarachnoid catheterization in rats. *Pharmacology, biochemistry, and behavior*. 1991; 38: 685-8.

3. **Julien N, Goffaux P, Arsenault P, Marchand S.** Widespread pain in fibromyalgia is related to a deficit of endogenous pain inhibition. *Pain*. 2005; 114: 295-302.

4. **Hayes AG, Sheehan MJ, Tyers MB.** Differential sensitivity of models of antinociception in the rat, mouse and guinea-pig to mu- and kappa-opioid receptor agonists. *British journal of pharmacology*. 1987; 91: 823-32.

5. **Wisniewski JR, Zougman A, Nagaraj N, Mann M.** Universal sample preparation method for proteome analysis. *Nature methods*. 2009; 6: 359-62.

6. **Tyanova S, Temu T, Cox J.** The MaxQuant computational platform for mass spectrometry-based shotgun proteomics. *Nature protocols*. 2016; 11: 2301-19.

7. **Li H, Xie H, Liu W, Hu R, Huang B, Tan YF, Xu K, Sheng ZF, Zhou HD, Wu XP, Luo XH.** A novel microRNA targeting HDAC5 regulates osteoblast differentiation in mice and contributes to primary osteoporosis in humans. *The Journal of clinical investigation*. 2009; 119: 3666-77.
